# Single-Step Phytate Flame-Retardant Coatings for Cotton, Polyester and Cotton/Polyester Blends

**DOI:** 10.3390/polym18070819

**Published:** 2026-03-27

**Authors:** Olga Zilke, Dennis Plohl, Martin Ploenißen, Alaa Salma, Dominic Danielsiek, Mariia Kuznetsova, Karlheinz Bretz, Philip Moerbitz, Jochen S. Gutmann, Klaus Opwis

**Affiliations:** 1 Deutsches Textilforschungszentrum Nord-West gGmbH, Adlerstr. 1, D-47798 Krefeld, Germany; dennis.plohl@dtnw.de (D.P.); martin.ploenissen@dtnw.de (M.P.); salma@dtnw.de (A.S.); danielsiek@dtnw.de (D.D.); jochen.gutmann@uni-due.de (J.S.G.); 2 Fraunhofer-Institut fuer Umwelt-, Sicherheits- und Energietechnik UMSICHT, Osterfelder Str. 3, D-46047 Oberhausen, Germany; mariia.kuznetsova@umsicht.fraunhofer.de (M.K.); karlheinz.bretz@umsicht.fraunhofer.de (K.B.); philip.moerbitz@umsicht.fraunhofer.de (P.M.); 3 Physical Chemistry & CENIDE, University Duisburg-Essen, Universitaetsstr. 2, D-45117 Essen, Germany

**Keywords:** flame retardancy, textiles, phytic acid, poly(vinylamine), chitosan

## Abstract

Scalable halogen-free flame-retardant textile finishes remain challenging, particularly regarding laundering durability and industrially viable processing. Here, two phytate flame retardants, poly(vinylammonium) phytate (PVAmPA, partly bio-based) and chitosan phytate (ChiPA, fully bio-based), were applied to cotton (CO), polyester (PET), and a CO/PET blend by a single-step, binder-assisted coating. Both coatings suppressed surface flaming in ISO 15025 on all substrates. Although laundering at 40 °C caused systematically higher wash-off for ChiPA, surface flame suppression was retained for most coated fabrics, with the exception of ChiPA on CO and PVAmPA on PET. Thermogravimetric analysis showed earlier decomposition and increased residue formation for both systems, with the residue at 700 °C increasing from 4.5% to 18.2% for CO_PVAmPA and from 4.5% to 15.2% for CO_ChiPA. In microscale combustion calorimetry, PVAmPA reduced the heat release capacity (HRC) from 251 to 168 J/(g·K) for CO/PET, whereas ChiPA showed its strongest effect on PET, reducing HRC from 413 to 222 J/(g·K). Gas-phase analyses indicated enhanced water release for both coatings and additional NH_3_ evolution for PVAmPA. Overall, binder-assisted, single-step phytate coatings provide a scalable route to halogen-free flame retardancy, with PVAmPA showing the most robust overall durability and ChiPA offering a fully bio-based alternative with strong substrate-dependent performance.

## 1. Introduction

Reducing fire risk in everyday materials is an essential element of modern safety strategies. Textiles and upholstered furnishings are particularly relevant because they are ubiquitous in dwellings and can support rapid flame growth. Recent fire statistics for England (year ending March 2025) [1] report that “textiles, upholstery and furnishings” were the items first ignited in 27% of primary dwelling fires and the materials mainly responsible for fire spread in 22% of cases while being associated with 49% of the corresponding fire-related fatalities.

Because textiles are polymeric and porous and offer a high specific surface area, they can ignite readily and contribute to fire development [2,3]. Flame-retardant (FR) finishes are therefore widely used, ranging from inorganic systems, intumescent coatings, and inherently non-combustible systems to highly efficient halogen-containing formulations [4]. However, increasing concerns regarding toxicity, persistence and bioaccumulation have accelerated the shift toward halogen-free FR concepts and more environmentally benign chemistries for polymeric materials [5,6].

**Figure 1:**
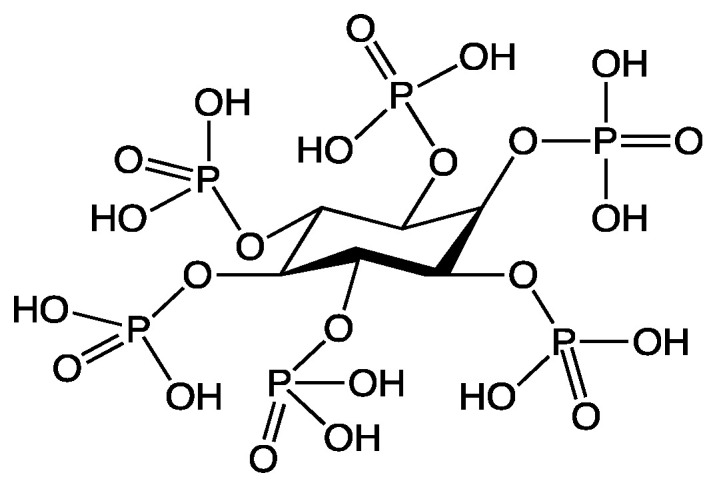
Chemical structure of phytic acid.

Among halogen-free approaches, advanced coating strategies such as layer-by-layer (LbL) assemblies [7,8,9,10] and sol–gel treatments [11,12,13] have demonstrated strong flame-retardant potential on textiles, including barrier formation and the promotion of condensed-phase residue. Nevertheless, these strategies are frequently limited by multi-step processing and challenges in translating laboratory protocols into industrially scalable finishing routes. In parallel, phosphorus-based flame retardants have gained increasing attention as halogen-free alternatives because of their strong condensed-phase activity and broad applicability. At the same time, phosphorus is a finite resource [14], and its long-term availability must be considered when assessing the sustainability of phosphorus-containing FR systems. In this context, phytic acid (PA) has emerged as a bio-derived [15,16,17,18], phosphorus-rich FR building block (Figure 1) that is available from plant resources [19,20] and can be sourced from cereal-derived side streams such as bran and other milling residues, which are frequently used in animal nutrition [19,20,21]. Because phytate strongly complexes nutritionally relevant minerals (e.g., Ca, Mg, Fe), it is considered anti-nutritive at high concentrations and is therefore commonly mitigated in feed by phytase-driven hydrolysis to improve mineral availability [20,21,22]. In this context, employing PA as a flame-retardant building block offers a valorization pathway for agro-industrial residues. In addition, bio-based cationic polymers such as chitosan, commonly produced by the deacetylation of chitin from crustacean shell waste or fungi [23,24,25], provide a nitrogen-containing counterpart that can be combined with PA to form flame-retardant P-N systems.

In such phosphorus–nitrogen systems, the two elements can act synergistically during thermal decomposition [26]. Phosphorus-containing species mainly promote condensed-phase activity by catalyzing dehydration and favoring the formation of a protective char layer, whereas nitrogen-containing components can support char expansion and stabilization and may additionally contribute to the dilution of combustible volatiles [27]. For cellulosic substrates in particular, P-N combinations are therefore often more effective than phosphorus alone because they promote the earlier formation of thermally insulating residue and reduce the release of flammable degradation products [28]. Against this background, chitosan is of interest not only as a bio-based cationic counterpart for phytic acid but also as a functional nitrogen-containing synergist in phytate-based FR systems.

Recent work has demonstrated that PA/chitosan assemblies can deliver very strong flame-retardant performance. Wang et al. [29] employed a stepwise dip-coating/self-assembly strategy combining PA and chitosan on cotton (CO) and reported LOI values up to 38.5% as well as pronounced reductions in the peak heat release rate (pkHRR) and total heat release (THR). Zhang et al. [30] developed a bio-derived intumescent flame-retardant finish for CO based on chitosan and PA using a dip–pad–dry approach that reduces the number of deposition steps compared with classical multilayer assemblies. The resulting coatings promoted earlier thermal activation and increased char formation in thermogravimetric analyses (TGA), lowered heat release parameters in microscale combustion calorimetry (MCC), and improved flammability performance (higher LOI and reduced burning rate). Durability was assessed by prolonged water exposure rather than standardized laundering, highlighting that the wash resistance of PA-based P-N systems remains a key practical limitation and motivates simplified, more robust finishing concepts. In our earlier work, we demonstrated that PA can be immobilized on CO via LbL assembly with polyvinylamine (PVAm) as a nitrogen-rich counterpart, yielding a partly bio-based P-N finish with strong reductions in the HRR and clear evidence for phosphate-driven dehydration and protective char formation. TGA/MCC and TGA–FTIR indicated an earlier onset of condensed-phase reactions, increased water release and a reduced evolution of toxic/flammable volatiles, consistent with a mechanism dominated by acid-catalyzed dehydration and barrier char formation [7]. Despite these advances, two barriers continue to limit the translation of PA-based concepts: the processing effort associated with multi-step deposition strategies and insufficient retention under realistic laundering conditions. This is mainly attributed to the high hydrophilicity/water solubility of phytate-based systems and to the fact that their fixation on textiles often relies predominantly on noncovalent interactions rather than on covalent anchoring. Therefore, improved durability generally requires additional insolubilization or crosslinking strategies.

Flame-retardant performance is also highly substrate-dependent. CO tends to decompose via dehydration and char formation [31], whereas polyester (PET) softens, melts and may drip, which can reduce burning in some configurations but may also promote fire spread [32]. CO/PET blend fabrics often exhibit a disproportionately high fire hazard due to the scaffolding effect [32,33]. While melt flow and dripping in 100% PET may remove fuel from the ignition zone, the charred cotton scaffold in blends can retain molten PET and thereby sustain burning, increasing the heat release compared with the single-component fabrics. This substrate-specific behavior motivates comparative studies across chemically distinct fabrics using identical finishing routes.

**Figure 2:**
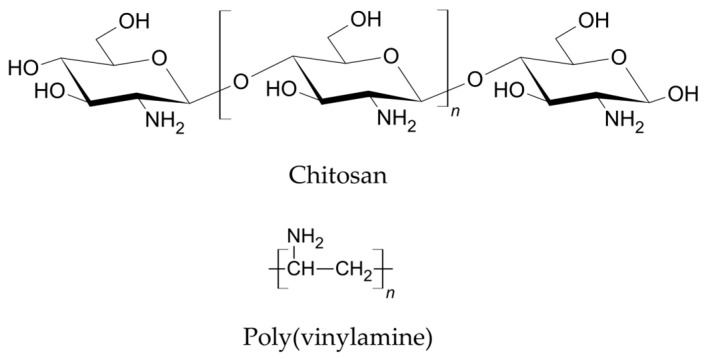
Chemical structures of chitosan and poly(vinylamine).

To address the need for scalable and durable halogen-free FR finishes across chemically distinct textile substrates, this study investigates two PA-based FR systems, applied in a single-step finishing process together with an acrylonitrile/acrylate binder. The FRs poly(vinylammonium) phytate (PVAmPA) and chitosan phytate (ChiPA) differ in their bio-based content (ChiPA being fully bio-based, while PVAmPA contains a synthetic PVAm component) and amine group density (Figure 2).

The treatments were applied to three industrially relevant fabrics, CO, PET, and a CO/PET blend, in a single-step, binder-assisted coating process to improve potential wash durability. The substrate-specific burning behavior, including the known challenges associated with polyester and the scaffolding effect in blends, was assessed. The performance of the finished fabrics was evaluated in terms of flame retardancy and wash durability. Flame-retardant behavior was assessed by standardized flammability testing, while washing resistance was examined after repeated laundering at 40 °C followed by renewed surface ignition testing. In parallel, the retention of the FR chemistry was quantified via elemental analysis (phosphorus and nitrogen), and thermal decomposition was characterized using thermogravimetric analyses (TGA), microscale combustion calorimetry (MCC), and pyrolysis–gas chromatography–mass spectrometry (Py-GC/MS). Finally, P/N loadings before and after burning were determined. Collectively, this study addresses whether a scalable single-step phytate finish can deliver flame suppression across distinct textile substrates while clarifying substrate- and formulation-dependent limitations in durability and decomposition behavior.

## 2. Materials and Methods

### 2.1. Flame Retardant Preparation

For the preparation of polyvinyl ammonium phytate (PVAmPA), 500 g of Lupamin 9095 (technical-grade polyvinylamine (PVAm), degree of hydrolysis > 90%, solid content 20–22%, BASF, Ludwigshafen am Rhein, Germany) was precipitated with 92.5 mL of 50% phytic acid solution (ca. 50% phytic acid in water, ca. 1.1 mol/L, TCI Europe N.V., Zwijndrecht, Belgium). For this purpose, Lupamin was diluted in a 10 L beaker to a 50 g/L solution and heated to 60 °C. The 92.5 mL phytic acid solution (equivalent to 67.2 g of phytic acid) was diluted to 925 mL to create a 5% (*v*/*v*) solution and then slowly added dropwise to the Lupamin solution under stirring until complete precipitation was achieved. The resulting polyvinyl ammonium phytate (PVAmPA) was subsequently filtered and washed with deionized water. The product was then dried in an oven at 80 °C and ground using a mill. The final product was sieved through a nylon mesh with a 100 µm pore size, yielding approximately 98 g (77%) of the product.

For the preparation of chitosan phytate (ChiPA), 120 g of ChiProPlant (99.9% water-soluble chitosan, 95% degree of deacetylation, ChiPro GmbH, Bremen, Germany) was dissolved in 7 L of preheated water (60 °C) in a 10 L beaker under stirring. Precipitation was carried out using a 5% (*v*/*v*) phytic acid solution. This PA solution was added dropwise to the chitosan solution under stirring until complete precipitation occurred. The ChiPA product was filtered and washed with deionized water. The product was dried in an oven at 80 °C, ground using a mill, and sieved through a nylon mesh with a 100 µm pore size. The yield of the product ranged between 151 and 158 g (>87%).

**Figure 3:**
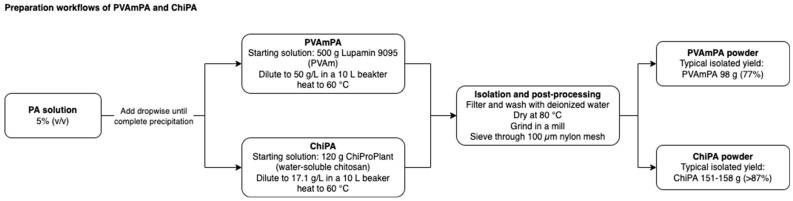
Schematic preparation workflow of PVAmPA and ChiPA.

The schematic preparation workflow of PVAmPA and ChiPA is shown in Figure 3.

### 2.2. Textile Coating

The coating process was performed using a Coatema coating system (Multifunctional Coating System—Basecoater, Coating Machinery GmbH (Coatema), Neuss, Germany). The formulations were prepared according to Table 1.

For this process, Lefasol 214/2 (acrylate copolymer dispersion with acrylonitrile, solid content (58 ± 1.5)%, Lefatex Chemie GmbH, Brueggen, Germany) was mixed with the appropriate amount of water and BYK 349 (polyether-modified siloxane, substrate wetting agent, BYK-Chemie GmbH, Wesel, Germany), and the FR was gradually added while stirring. The formulation was then homogenized for an additional 10 min.

The following commercial fabrics were coated with the formulations containing PVAmPA and ChiPA:

- A CO fabric (area weight 170 g/m^2^; plain weave, Wfk Testgewebe GmbH, Brueggen, Germany);
- A PET fabric (area weight 170 g/m^2^, Wfk Testgewebe GmbH, Brueggen, Germany);
- A 50/50 CO/PET core yarn fabric (twill 2/1, PET core, CO shell, 170 g/m^2^, camouflage, Bluecher GmbH, Erkrath, Germany).

The fabrics were coated using a doctor blade (PA-2328, 0–3800 µm, BYK-Gardner GmbH, Geretsried, Germany) with a 200 µm gap. The coated textile was subsequently pre-dried at 60 °C and then cured in an oven at 130 °C for at least 1 min. Finally, the textile was rinsed for 1 min with deionized water and dried at 60 °C.

### 2.3. Analyses

The mass add-on A (%) was determined with

A = (m_1_ − m_0_)/m_0_ × 100%, (1)

where m_1_ (g) represents the mass after treatment, and m_0_ (g) represents the mass before treatment.

The coating thickness was measured by the use of a thickness gauge (Rainbow, Karl Schroeder KG, Weinheim, Germany) relative to the uncoated fabric (*n* = 10) under a measuring pressure of 1.00 kPa and a contact area of 2000 mm^2^.

The pyrolysis behavior of PVAmPA, ChiPA, Lupamin, chitosan, and PA, as well as the pristine and coated fabrics, was examined using a Discovery TGA55 (TA Instruments, Huellhorst, Germany). The samples were pre-dried for 48 h at 60 °C, and 10 mg ± 1 mg of each sample was weighed and analyzed. Measurements were conducted under nitrogen (90 mL/min) in a ceramic crucible. The system was first allowed to equilibrate at 40 °C and isothermally held at this temperature for 5 min. Samples were then heated from 40 °C at a rate of 20 K/min to 800 °C where it was held for 10 min before cooling down. Decomposition products were identified using literature data [34], SpectraBase [35] and the reference spectra of low-molecular-weight gaseous compounds reported by Ngohang et al. [36]. Microscale combustion calorimetry (MCC) was carried out in triplicate according to ASTM D7309-13 [37] using an FAA Micro Calorimeter (Fire Testing Technology, East Grinstead, UK). Samples were pyrolyzed under nitrogen (heating rate 1 K/s to 750 °C) and the volatiles oxidized in an O_2_/N_2_ (20:80) mixture at 900 °C. Peak deconvolution and analysis were performed using Microcal Origin 2018b Professional. The maxima of the individual peaks were summed to obtain the heat release capacity (HRC), while the peak areas (peak heat release, pkHR) were integrated and summed to yield the total heat release (THR).

The nitrogen content of the FRs and the coated textiles was determined through the potentiometric titration of Kjeldahl nitrogen. For this, approximately 200 mg of the samples was digested in 20 mL of H_2_SO_4_ (ROTIPURAN 98%, Carl Roth, Karlsruhe, Germany) with the addition of two Kjeldahl tablets (350–380 °C, ≥120 min). For the subsequent steam distillation, 50 mL of NaOH (50%, extra pure, Carl Roth, Karlsruhe, Germany) was added to the digested sample, and the resulting ammonia was distilled into an aqueous H_3_BO_3_ (≥99.5%, Ph.Eur., USP, BP, Bernd Kraft GmbH, Duisburg, Germany) absorber solution. The ammonium ions were then titrated potentiometrically with HCl (0.05 mol/L, volumetric solution, Carl Roth, Karlsruhe, Germany). The nitrogen mass fraction *w*(N) was calculated. For the determination of the phosphorus content, 100–200 mg of the samples was digested in 8 mL of HNO_3_ (69%, supra-quality ROTIPURAN, Carl Roth, Karlsruhe, Germany) in a microwave at 190 °C. After cooling, the samples were transferred to a volumetric flask and stored overnight in a refrigerator. The solution, brought to room temperature, was then diluted with water and membrane-filtered. Subsequently, elemental analyses were performed using ICP-OES.

The limiting oxygen index (LOI) was determined on one 5 cm × 10 cm specimen in accordance with DIN EN ISO 4589-2 [38], with the ignition flame applied to the upper edge. Flame-retardant performance was further evaluated by surface ignition tests according to DIN EN ISO 15025 [39], using a flame application time of 10 s. Pass/fail criteria followed DIN EN ISO 11611 [40], requiring no hole formation, no dripping, and afterflame and afterglow times ≤ 2 s.

Washing resistance was investigated using a Linitester (Heraeus-Original, Hanau, Germany), a mechanical washing device of the type used for DIN EN ISO 105-C06 [41] testing. Samples were washed at 40 °C for 30 min using ECE reference detergent without optical brightener (4 g/L). Up to ten washing cycles were performed. After washing, the samples were subjected to surface ignition tests according to DIN EN ISO 15025. The add-on and the phosphorus (ICP-OES) and nitrogen (Kjeldahl) contents were determined.

Pristine and coated fabrics were subjected to pyrolysis at 700 °C for 1 min in a sample tube placed in the thermal desorption system (TDS). Gas chromatography–mass spectrometry (GC–MS) conditions were as follows: a cooled injection system with an initial temperature of −50 °C, ramped at 12 °C/s to 300 °C; an HP Ultra 2 capillary column with helium as the carrier gas, oven temperature programmed from 50 to 300 °C at 10 °C/min; electron ionization at 70 eV, with positive ions recorded over *m*/*z* 35–620. Chromatograms were processed using OpenChrom Lablicate Edition (1.4.0.202103172155) [42], and peaks were assigned with the aid of the NIST Standard Reference Database 69 [43], MassBank [44] and SpectraBase [35].

For elemental analysis, five circular specimens (diameter 4 cm) were exposed to a flame according to DIN EN ISO 15025; burned and corresponding unburned samples (diameter 1.6 cm) were then analyzed for phosphorus by ICP-OES and for nitrogen by Kjeldahl analyses. In addition, the remaining residue after flame exposure was quantified gravimetrically. Phosphorus and nitrogen surface densities were calculated on the basis of the sample area, and mean values and standard deviations were derived from five replicates.

**Figure 4:**
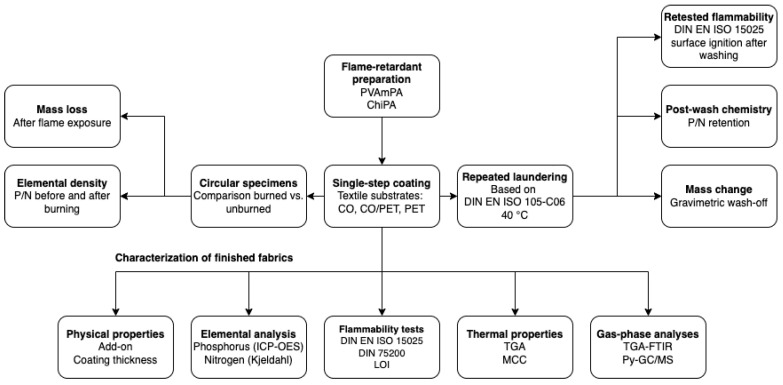
Schematic overview of experimental workflow [38,39,45].

A schematic overview of the experimental workflow is shown in Figure 4.

## 3. Results and Discussion

### 3.1. Flame Retardants

#### 3.1.1. Characterization

Phosphorus-based FRs primarily act in the condensed phase. Upon heating, phosphate-containing species can generate phosphoric acid and related acids through endothermic reactions, which promote dehydration and the formation of a thin, glass-like layer which reduces oxygen diffusion, the release of decomposition products, and heat transfer [46]. When combined with other flame-retardant elements, such as nitrogen, synergistic effects have been reported for certain systems, which are frequently attributed to the formation of polymeric P-N-containing species, driven by the nucleophilic attack of nitrogen on phosphate groups. Moreover, P-N bonds are more electrophilic, which enhances the phosphorylation of primary C(6)-hydroxylic groups in CO [47,48].

Two different FRs were produced from commercial PA in combination with synthetic PVAm and bio-based chitosan, yielding PVAmPA and ChiPA, respectively.

PVAm provides a high density of primary amine functionalities (and thus nitrogen) that can form strong ionic interactions with polyanions such as PA, whereas chitosan is a biocompatible polysaccharide containing primary amine groups (depending on the degree of deacetylation) and can contribute to condensed-phase char formation. Chitosan is biodegradable and is therefore widely regarded as an environmentally benign material. As it is derived from chitin, a naturally abundant renewable resource, chitosan is commonly considered a sustainable bio-based building block [23].

PVAmPA and ChiPA were characterized in terms of phosphorus and nitrogen contents and the corresponding molar P/N ratios in the product (Table 2).

The higher phosphorus and nitrogen mass fractions in PVAmPA compared with ChiPA can be explained by the different repeat unit masses and amine site densities of the two polymers. PVAm consists of a small repeat unit with one amine functionality, which provides a high number of protonatable amine groups per gram and allows for the incorporation of a relatively large amount of phytate per unit mass of the complex. Chitosan, in contrast, has a much larger saccharide repeat unit and therefore a lower number of amine groups per gram, so the resulting phytate complex contains a higher fraction of polymer backbone mass, which lowers the mass fractions *w*(P) and *w*(N).

On a molar basis, the measured P/N ratios indicate that not every phosphate group is paired with a nitrogen in ChiPA. This is consistent with the partial protonation of phosphate groups (depending on pH and counterions) and with steric constraints in the chitosan matrix, which can limit the extent of ionic association between phytate groups and amines.

In conclusion, PVAmPA, with its higher levels of both phosphorus and nitrogen, is expected to provide superior flame retardancy compared to ChiPA.

#### 3.1.2. Thermal Properties

**Figure 5:**
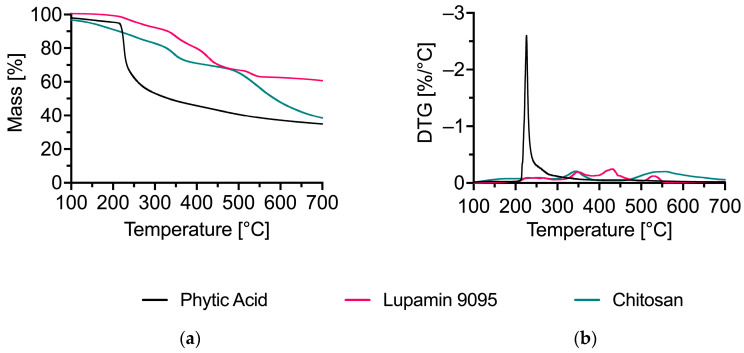
(**a**) Thermogravimetric analyses (TGA); (**b**) the differential TG (DTG) curves of phytic acid, Lupamin 9095, and chitosan measured under nitrogen at a heating rate of 20 K/min.

The raw materials used for the production of PVAmPA and ChiPA were analyzed by TGA. The TGA and derivative thermogravimetric (DTG) curves are shown in Figure 5, while the corresponding data are summarized in Table 3.

PA exhibits a pronounced initial mass loss despite pre-drying, consistent with its hygroscopic character and the release of strongly bound water, resulting in a high mass loss of 5% of the initial mass at 146 °C (T_Δ5%_). PA decomposes gradually over a broad temperature range and shows two distinct DTG maxima at 335 and 559 °C, leaving a substantial residue of 38.5% at 700 °C (Res_700°C_). Chitosan shows a more clearly defined main decomposition step with a DTG maximum at 226 °C and a residue of 34.9% at 700 °C.

**Figure 6:**
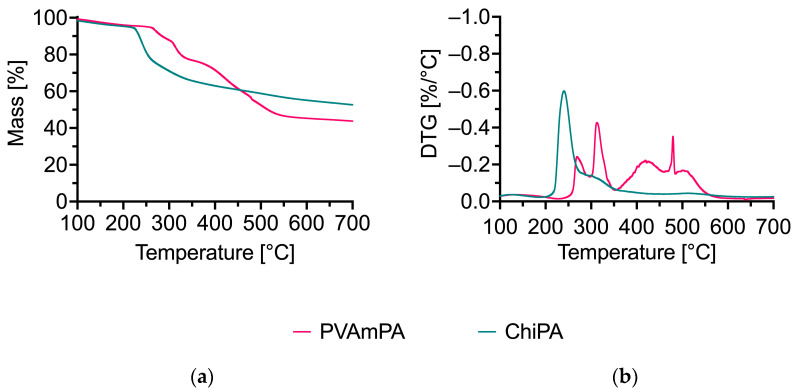
(**a**) Thermogravimetric analyses (TGA); (**b**) the differential TG (DTG) curves of PVAmPA and ChiPA measured under nitrogen at a heating rate of 20 K/min.

When chitosan is combined with phytic acid in ChiPA, the intensity of this peak diminishes significantly (Figure 6). ChiPA exhibits a considerably higher residual mass at both 240 °C (86.7%) and 700 °C (52.6%) compared to pure chitosan. The increased char yield and altered decomposition profile indicate a strong interaction between chitosan and phytate species, which favors condensed-phase residue formation.

Lupamin 9095 displays multiple decomposition steps and a high residue at 700 °C (60.7%), which is consistent with the presence of non-volatile inorganic constituents (e.g., salts) in the technical-grade material. In PVAmPA, the decomposition of PVAm is slightly accelerated by the presence of phytic acid. At 254 °C, 5% of the mass has already been released, and the decomposition peaks were shifted toward lower temperatures. This catalytic decomposition driven by PA proves especially beneficial in flame retardancy. The early onset of decomposition may lead to the formation of inert structures on textile surfaces, which are able to effectively protect the material from further pyrolysis. At the same time, the lower residue of PVAmPA (43.8% at 700 °C) compared with Lupamin is consistent with the partial removal of inorganic constituents during precipitation and washing. Overall, both phytate complexes (PVAmPA and ChiPA) show increased residue formation compared to their parent polymers, supporting their potential for condensed-phase flame-retardant action.

### 3.2. Flame-Retardant Coating

#### 3.2.1. Characterization

**Figure 7:**
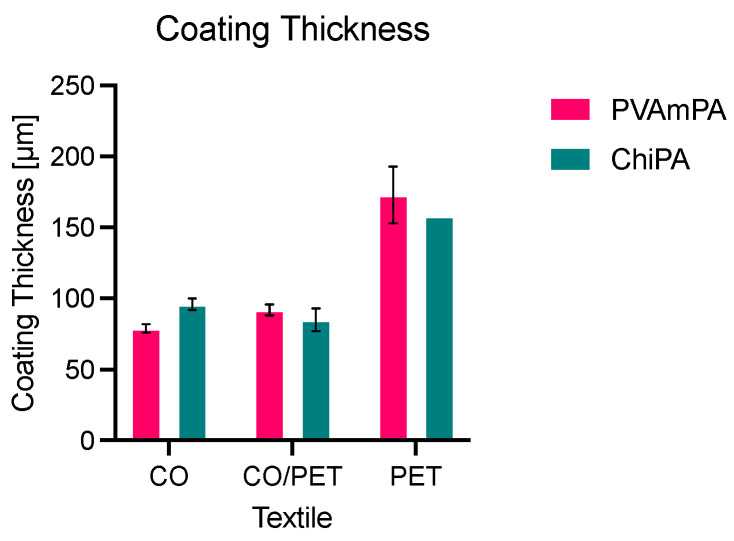
Coating thickness of coated CO, CO/PET, and PET.

The thickness of the flame-retardant coating on CO, CO/PET, and PET was determined relative to the corresponding uncoated fabrics (Figure 7).

With a theoretical wet film thickness of 200 µm, dry coating thicknesses of approximately 75–100 µm were obtained on CO and CO/PET, whereas PET exhibited substantially higher thickness values, reaching up to ~175 µm.

This difference is consistent with substrate-dependent coating formation. CO and CO/PET are more readily wetted and allow for the partial penetration of the formulation into the porous fiber, which reduces the apparent thickness of the surface layer after drying and curing. In contrast, the hydrophobic PET fabric shows limited penetration and forms a more pronounced surface layer, resulting in higher measured thickness values.

**Figure 8:**
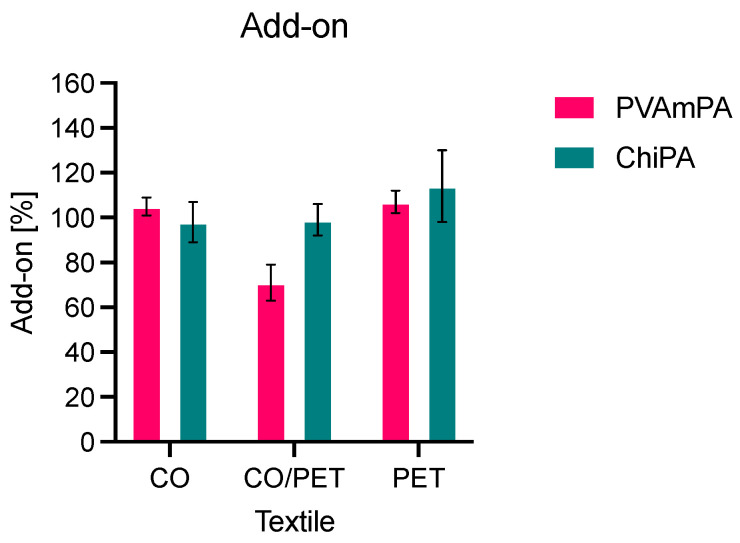
Coating add-on of CO, CO/PET, and PET.

Although the add-on values were comparable across the three fabric types (Figure 8), the coating thickness data indicate that the distribution of the coating between fiber interior and surface layer differs between the natural and synthetic substrates, which can be expected to influence subsequent fire performance and wash durability.

A more pronounced surface layer, as observed for PET, may enhance barrier effects by limiting heat and oxygen transfer and by restricting the release of flammable volatiles. In CO and CO/PET, the partial penetration of the formulation into the porous fiber assembly is expected to increase the interfacial contact area between the FR species and the substrate, which may promote dehydration/char formation on cellulose fibers and improve the mechanical anchoring of the finish, potentially benefiting washing durability.

#### 3.2.2. Washing Stability

**Figure 9:**
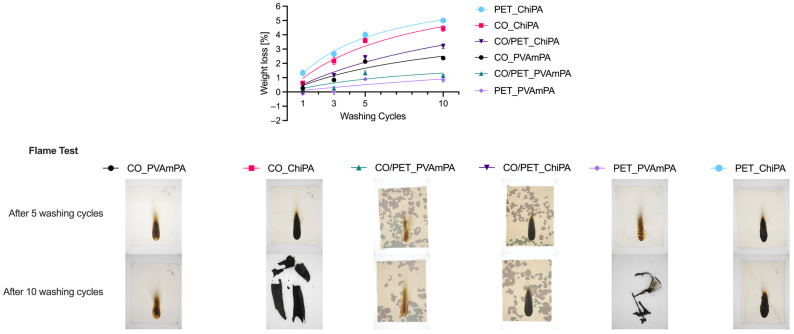
Weight loss curves of coated CO, CO/PET, and PET after one, three, five and ten washing cycles at 40 °C and flame test results after five and ten washing cycles according to DIN EN ISO 15025 (surface ignition).

Washing stability was evaluated after one, three, five and ten washing cycles at 40 °C (Figure 9). After ten wash cycles, the cumulative mass loss remained within ~5%, meaning that >95% of the mass was retained. For all coated fabrics, an increase in weight loss was observed with an increasing number of washing cycles, indicating the gradual removal of coating material during repeated laundering. The extent of mass loss varies between the two FRs. In all three textile substrates, the ChiPA-finished fabrics showed the highest weight losses, indicating the lower resistance of this system against repeated laundering compared with PVAmPA. This difference became more pronounced after five and ten washing cycles, suggesting that a larger fraction of the ChiPA-based finish is only weakly fixed and is progressively removed during washing.

Substrate dependence is plausibly influenced by the binder, since wetting, penetration and film formation differ between CO and PET-based textiles, thereby affecting coating anchoring and wash-off. The acrylate binder with acrylonitrile provides film continuity and can physically entrap the FRs within the polymer matrix., but it may also contribute to fixation via noncovalent interactions. In particular, carboxylic acid functionalities may form ionic pairs with protonated amines (PVAm, chitosan), which is expected to influence resistance to aqueous extraction. Additional, weaker hydrogen-bonding interactions with hydroxyl-rich components (cellulose, chitosan) may occur. On this basis, PVAmPA, with higher amine charge density and steric accessibility, may interact more efficiently with the binder network, consistent with lower overall gravimetric wash-off. In contrast, ChiPA coatings can exhibit higher gravimetric wash-off but comparatively small P/N changes on PET and CO/PET, which is consistent with the preferential loss of binder-dominated material. Although covalent coupling between binder carboxyl groups and hydroxyl or amine functionalities (ester or amide formation) is in principle possible, the applied curing conditions (130 °C) are more consistent with predominantly noncovalent fixation [49].

**Figure 10:**
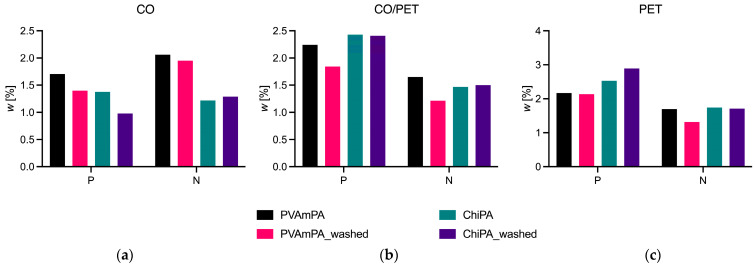
(**a**–**c**) Phosphorus and nitrogen mass fractions before and after 10 washing cycles.

To relate the observed weight loss to chemical retention, the phosphorus and nitrogen mass fractions of the coated fabrics were quantified before and after 10 washing cycles (Figure 10). Overall, laundering reduced the elemental loadings for most combinations, confirming that the coating components containing P and N are progressively removed during washing. For CO, both *w*(P) and *w*(N) decreased after washing for PVAmPA and ChiPA, with a more pronounced reduction in *w*(P) for ChiPA. For the CO/PET blend, PVAmPA showed a clear decrease in both *w*(P) and *w*(N), whereas ChiPA exhibited essentially unchanged phosphorus mass fractions and only minor changes in *w*(N), indicating a higher retention of the phosphorus-containing fraction in this substrate/FR combination. For PET, *w*(N) decreased substantially for PVAmPA after washing, while ChiPA showed comparatively stable *w*(N) and no decrease in phosphorus. Taken together, the elemental analysis corroborates the washing stability trends from the weight loss data and the flame tests and demonstrates that the retention of P/N-containing species is strongly dependent on both the textile substrate and the FR system. In general, improving laundering resistance often requires stronger fixation mechanisms, for example, through binder optimization, crosslinking approaches, or covalent grafting strategies [50,51,52].

#### 3.2.3. Thermal Properties

**Figure 11:**
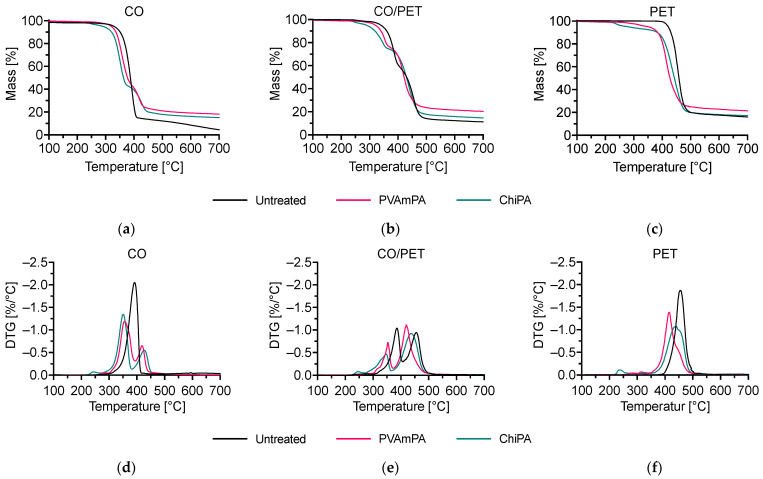
(**a**–**c**) Thermogravimetric analyses (TGA) and (**d**–**f**) differential TG (DTG) curves of CO, CO/PET, and PET coated with PVAmPA and ChiPA, measured under nitrogen at a heating rate of 20 K/min.

Figure 11 and Table 4 summarize the thermal degradation behavior of the untreated and finished fabrics under nitrogen.

For all three substrates, the application of PVAmPA and ChiPA shifts the onset of mass loss to lower temperatures (T_Δ5%_), which is consistent with an early decomposition of the coating system and/or acid-catalyzed reactions initiated by phosphate species [53]. In addition to the possible phosphorylation reactions of CO, the phosphoric acid released upon heating can catalyze dehydration and promote char formation in the condensed phase, thereby altering the early degradation pathway [53]. This effect is the most pronounced for PET, where T_Δ5%_ decreases from 421 °C (untreated) to 350 °C (PVAmPA) and 280 °C (ChiPA), indicating that the initial mass loss is dominated by the finish rather than the PET backbone itself.

Both finishes increase the residue at 700 °C compared with the corresponding untreated fabrics, confirming enhanced condensed-phase residue formation. For CO, the residue increases from 4.5% to 18.2% (PVAmPA) and 15.2% (ChiPA), for the CO/PET blend from 11.3% to 20.4% (PVAmPA) and 14.6% (ChiPA), and for PET from 16.0% to 21.5% (PVAmPA) and 17.3% (ChiPA). PVAmPA consistently yields the highest final residue, while ChiPA induces an even earlier onset of mass loss but leads to a smaller residue increase, particularly on PET. Overall, these trends indicate that both systems promote condensed-phase pathways, with PVAmPA showing a stronger contribution to char/residue formation across all three textile types. The high char yield indicates that PVAmPA effectively catalyzes char formation, likely through phosphorus-driven dehydration reactions.

**Figure 12:**
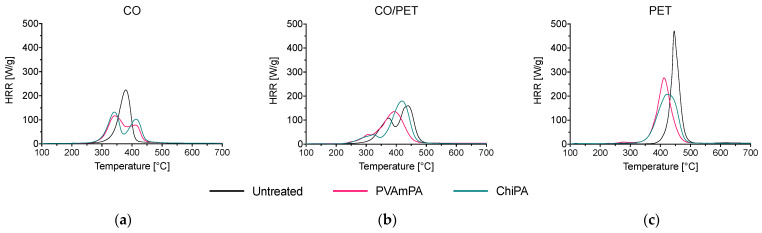
Microscale combustion calorimetry (MCC) measurements of untreated and coated (**a**) CO, (**b**) CO/PET, and (**c**) PET.

The MCC results provide a clear indication of the FRs’ effectiveness in reducing the heat release during combustion (Figure 12, Table 5 and Table A1).

For CO, PVAmPA reduces the heat release capacity (HRC) from 208 to 173 J/(g·K), whereas ChiPA results in an HRC of 225 J/(g·K), slightly higher than that of the untreated fabric. The total heat release (THR) remains essentially unchanged for CO (≈10–11 kJ/g). Hence, only PVAmPA reduces peak heat release intensity under MCC conditions, despite both finishes increasing TGA residue.

For the CO/PET blend, both finishes reduce HRC, with PVAmPA showing the strongest effect (from 251 to 168 J/(g·K)) and ChiPA providing a moderate reduction (from 251 to 218 J/(g·K)). In addition, THR decreases from 14.6 to 12.8 kJ/g for PVAmPA and to 14.0 kJ/g for ChiPA, suggesting that PVAmPA more effectively limits the overall combustible volatile output in this mixed substrate system. The peak HRR (T_pkHRR_) is also shifted to lower temperatures and shows multi-step behavior, consistent with the early degradation of the finish and subsequent decomposition of the textile matrix.

For PET, both finishes substantially reduce HRC from 413 to 251 J/(g·K) (PVAmPA) and 222 J/(g·K) (ChiPA), demonstrating a strong reduction in peak heat release. However, THR for PET remains essentially unchanged for PVAmPA (from 14.8 to 14.6 kJ/g) and increases for ChiPA (from 14.8 to 16.2 kJ/g). This combination (lower HRC but unchanged/increased THR) suggests that while the finishes can suppress peak heat release, the total amount of combustible material released and oxidized is not necessarily reduced under MCC conditions, particularly for ChiPA on PET. Therefore, both HRC and THR should be considered together when ranking performance.

#### 3.2.4. Flame Tests

Flame-retardant performance was assessed using standardized ignition and flammability tests, including DIN EN ISO 15025 (protective clothing), DIN 75200 (automotive standard) [45], and DIN EN ISO 4589-2 (LOI determination). The results provide a comparative evaluation of untreated fabrics and fabrics finished with PVAmPA or ChiPA.

**Figure 13:**
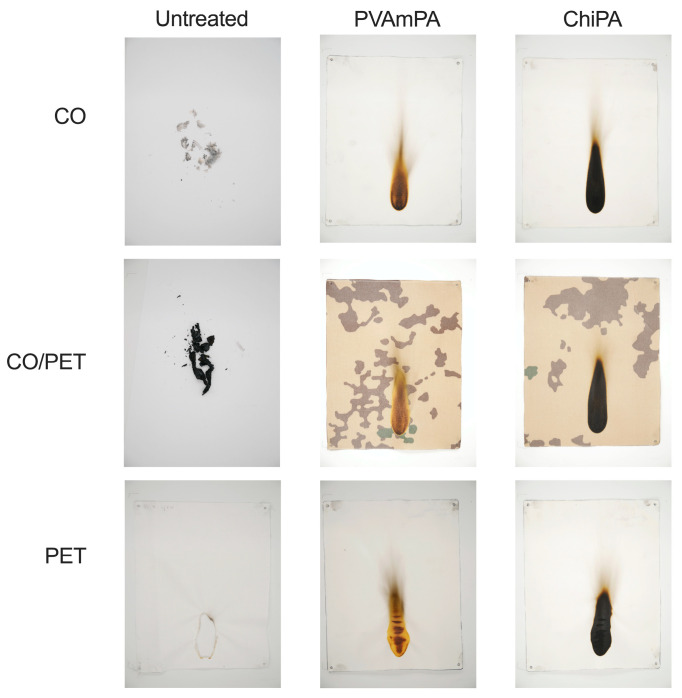
Results of surface ignition test according to DIN EN ISO 15025.

All untreated fabrics failed the ISO 15025 surface ignition test (Figure 13). CO-containing substrates (CO and CO/PET) exhibited extensive burning and loss of structural integrity, confirming their high flammability under these conditions. The untreated PET fabric also failed due to thermoplastic melting and hole formation, accompanied by dripping. In contrast, all fabrics finished with PVAmPA or ChiPA met the ISO 15025 pass criteria for surface ignition, indicating that both coatings effectively suppressed flame spread at the surface.

**Figure 14:**
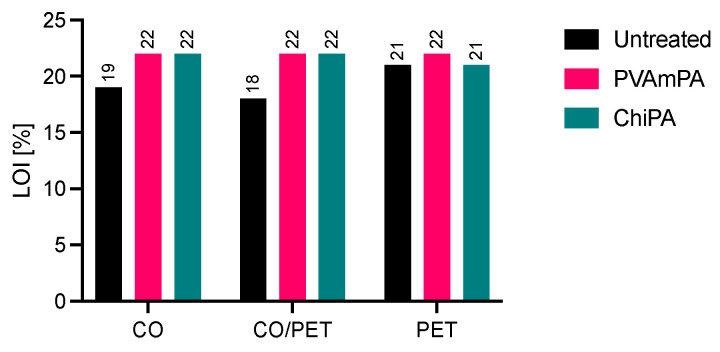
Limiting oxygen index (LOI) of treated and untreated textiles.

The LOI test evaluates the minimum oxygen concentration required to sustain combustion. The results are shown in Figure 14. The untreated textiles exhibited low LOI values, consistent with high flammability. These fabrics would readily ignite and sustain combustion in an atmosphere containing normal oxygen levels (around 21% oxygen in air). Both PVAmPA and ChiPA increased the LOI to ≥21% for CO and CO/PET and for PET finished with PVAmPA, whereas PET finished with ChiPA did not reach the same level. However, since the LOI values are close to the oxygen content in ambient air, the treated textiles remain flammable under the test conditions. This means that while the coatings improve fire resistance, the materials could still ignite and burn in typical atmospheric conditions without additional protective measures.

For the intended application in vehicle interiors, burning rates were determined according to DIN 75200, testing both the coated and uncoated sides of the fabrics (Table 6), as the orientation in the final application has not yet been defined. The results were benchmarked against the commonly applied acceptance criterion of 102 mm/min (FMVSS 302). Untreated textiles, except PET, failed the test, emphasizing the necessity of flame-retardant treatments for fire safety. PVAmPA showed consistently lower burning rates than ChiPA on CO and CO/PET, and the coated, flame-exposed side of PVAmPA-treated CO and CO/PET exhibited complete flame suppression (0 mm/min). ChiPA also reduced burning rates compared with the untreated fabrics but remained higher than those of PVAmPA on CO and CO/PET. For PET, both finishes achieved 0 mm/min on the coated side, while the uncoated side showed marked differences (PVAmPA: 0 mm/min; ChiPA: 128 mm/min), highlighting the importance of coating orientation and finish retention on this substrate.

Overall, the comparative results indicate that both finishes provide effective surface flame suppression under ISO 15025, while PVAmPA delivers the most robust performance across substrates in the DIN 75200 burning rate test. ChiPA shows a stronger dependence on substrate and orientation, which may require optimization for PET-containing constructions.

#### 3.2.5. Gas-Phase Analyses

**Figure 15:**
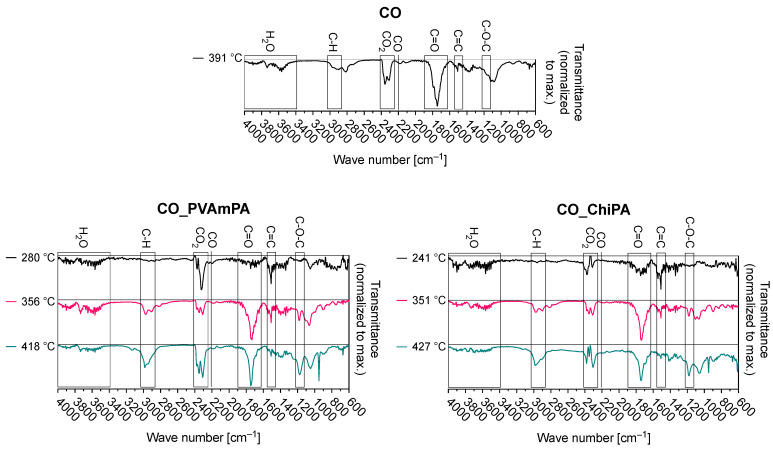
Baseline-corrected and normalized FTIR spectra of TGA peak maxima, measured under nitrogen at 20 K/min.

The TGA–FTIR measurements provide qualitative information on the evolution of the volatile decomposition products released during thermal treatment under nitrogen. Figure 15 shows baseline-corrected and normalized FTIR spectra extracted at the maxima of selected Gram–Schmidt peaks for the CO/PET blend. Spectra for the remaining samples are provided in the Supporting Information (Figure S1).

The untreated CO-containing fabric exhibits bands characteristic of cellulose pyrolysis, including strong absorptions in the O–H stretching region (≈3700–3100 cm^−1^, H_2_O), CO_2_ asymmetric stretching (≈2350 cm^−1^), carbonyl-containing volatiles (≈1800–1650 cm^−1^), and C–O stretching vibrations in the 1200–900 cm^−1^ region that are commonly associated with carbohydrate-derived species. Untreated PET shows dominant CO_2_/CO features and additional absorptions in regions typically assigned to ester- and aromatic-related vibrations (e.g., ~1600–1500 cm^−1^ for aromatic rings and 1100 cm^−1^ for ester decomposition products), consistent with polyester backbone decomposition. The CO/PET blend combines these decomposition products, indicating concurrent contributions from cellulose- and PET-derived volatiles.

After flame-retardant finishing, an increased intensity of O–H stretching bands (3700–3400 cm^−1^) at early decomposition stages suggests an enhanced release of water and/or other hydroxyl-containing volatiles. This observation is consistent with condensed-phase pathways involving dehydration and early residue formation promoted by phosphate species [47,54]. Changes in the 3200–3000 cm^−1^ region are assigned to alkenyl or aromatic =C–H stretching vibrations, and an increased contribution here can indicate an enhanced formation of unsaturated and aromatic structures during acid-catalyzed dehydration and aromatization processes that accompany char development [27]. CO_2_, a non-combustible gas, is consistently observed across all samples and treatments as a natural by-product of thermal degradation.

PVAmPA-treated samples, particularly PET and CO/PET, show the presence of NH_3_ in the gas phase (1000–900 cm^−1^). This is attributed to the decomposition of the nitrogen-rich PVAm structure. NH_3_ acts as a flame inhibitor in the gas phase, diluting combustible volatiles and further enhancing flame retardancy. ChiPA-treated samples do not exhibit NH_3_ release, reflecting the different decomposition chemistry of chitosan, which primarily releases non-nitrogenous volatiles. In addition, differences in the fingerprint region (1200–900 cm^−1^) may indicate changes in the relative contribution of carbohydrate-derived volatiles and phosphate-containing species.

**Figure 16:**
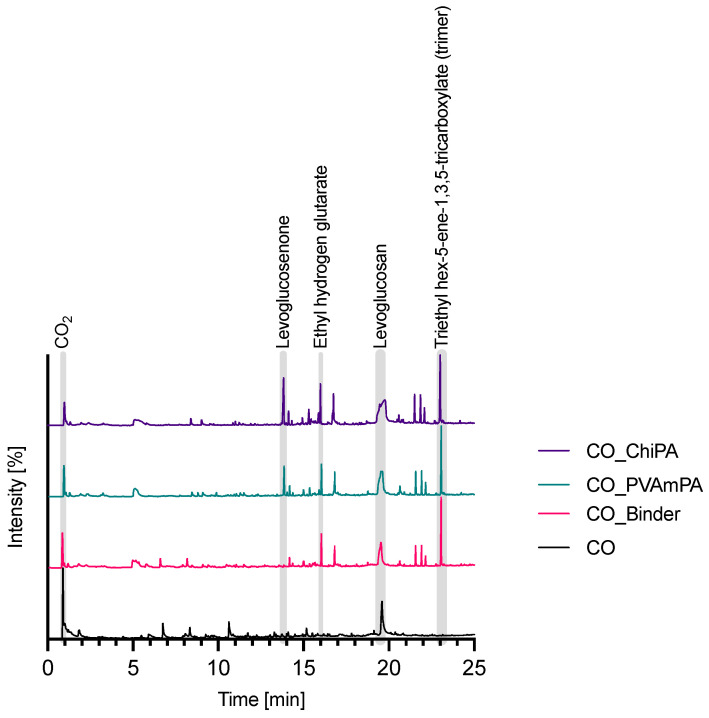
Total ion chromatograms (TICs) from Py-GC/MS analyses of CO-based textiles with and without flame-retardant treatment.

Py-GC/MS analyses provide detailed molecular-level information on the volatile products released during the thermal decomposition of treated and untreated textiles. Figure 16 shows the total ion chromatograms (TICs) of CO-based samples with and without flame-retardant treatment. Additional data for PET and CO/PET is available in the Supporting Information (Figures S3 and S4). In addition, overview tables (Tables S1–S3) and a heat map (Figure S2) summarize the compounds detected and not detected across the sample set.

For untreated CO, typical cellulose-derived oxygenates were observed, including furanic compounds and anhydrosugars such as levoglucosan, consistent with cellulose depolymerization and dehydration reactions [28,55,56]. Binder-treated CO additionally showed coating-related products, including ethyl acrylate and glutarate-type derivatives, indicating contributions from the acrylonitrile/acrylate binder system. In both FR-treated CO samples, levoglucosan and levoglucosenone were detected. Since levoglucosenone can form via the secondary dehydration/fragmentation of levoglucosan, its presence is consistent with the enhanced dehydration pathways promoted by phosphate-containing species during pyrolysis. This promotes char formation by facilitating the removal of water, which contributes to the flame-retardant effect. The increased release of water absorbs heat due to its high heat capacity and dilutes the concentration of combustible volatiles in the gas phase [57]. The formation of nitrogen-containing compounds such as 4(1H)-pyrimidinone (CO_PVAmPA) and glutarimide (CO_ChiPA) suggests the active participation of the nitrogen content from the FRs during decomposition. Additionally, the acrylonitrile component within the binder might undergo altered degradation in the presence of the FRs.

For PET, the untreated fabric released a set of characteristic aromatic and ester-related products typically reported for polyester pyrolysis, including benzene and vinyl/terephthalate-derived fragments [58]. In contrast to the CO-based systems, the binder-treated PET did not show additional nitrogen-containing products, suggesting that the binder contribution is dominated by acrylic/ester fragments rather than nitrogenous volatiles under these conditions. After FR finishing, additional low-molecular aromatic products (e.g., styrene, acetophenone) and nitrogen-containing species (e.g., pyridine) were detected, while several higher-molecular terephthalate-related fragments (such as ethylene dibenzoate, (1,1′-biphenyl)-2,2′-dicarboxaldehyde, and 1,3-benzenedicarboxylic acid, diethyl ester) were less prominent, indicating a shift in the fragmentation pattern in the presence of phosphate- and nitrogen-containing components.

The CO/PET blend exhibited a mixed product spectrum, including cellulose- and polyester-related marker compounds (e.g., levoglucosan and divinyl terephthalate). Notably, levoglucosenone remained detectable, whereas several marker compounds prominent in neat CO (1,4:3,6-dianhydro-α-D-glucopyranose, 5-hydroxymethylfurfural, 1,6-anhydro-β-D-glucofuranose, and ethyl hydrogen glutarate) and some PET-related alcohols (e.g., 1-hexanol and 2-ethyl-1-hexanol) were not observed. This can be rationalized by matrix and secondary reaction effects in the blend. First, because both fiber fractions are reduced compared with the pure substrates, the corresponding primary pyrolysis products are generated and released at substantially lower concentrations and may therefore fall below the detection limit. Second, the presence of molten PET together with acidic phosphate species can create a reactive condensed phase that promotes secondary dehydration, recombination and charring reactions, thereby favoring more condensed products (e.g., levoglucosenone and char-related fragments), suppressing several typical CO and PET marker volatiles.

Notably, ethyl acrylate and pentanedioic acid, 2-methylene-, diethyl ester were found in all samples treated with the binder. These compounds might be attributed to the binder itself.

Overall, TGA–FTIR revealed substrate-specific volatile profiles under nitrogen, with CO showing typical cellulose-derived bands (H_2_O, CO_2_, carbonyl and carbohydrate-related vibrations), PET exhibiting mainly CO_2_/CO and ester/aromatic features, and the CO/PET blend combining both signatures. After PVAmPA or ChiPA finishing, the early evolution of O–H-containing volatiles increased, consistent with phosphate-promoted dehydration pathways and an enhanced tendency toward condensed-phase residue formation. In PVAmPA-treated PET and CO/PET, additional absorptions attributable to nitrogen-containing volatiles were observed, whereas ChiPA-treated samples showed a different gas-phase composition without comparable features.

Py-GC/MS complemented these findings at the molecular level. CO released typical anhydrosugars and furanic oxygenates (e.g., levoglucosan and furfural derivatives), while binder-containing samples additionally produced characteristic acrylic/ester fragments (e.g., ethyl acrylate and 2-methylene-pentanedioic acid diethyl ester). FR finishing promoted dehydration-related products such as levoglucosenone and, for PVAmPA, a broader set of nitrogen-containing volatiles. PET exhibited the expected terephthalate-/aromatic-derived fragments in the untreated state, whereas FR finishing shifted the product pattern toward smaller aromatics and N-containing species and reduced the prominence of some higher-molecular fragments. In the CO/PET blend, several classical CO and PET marker compounds were suppressed, which is consistent with lower release concentrations and matrix-driven secondary reactions in a reactive condensed phase.

#### 3.2.6. Condensed-Phase Analyses

**Figure 17:**
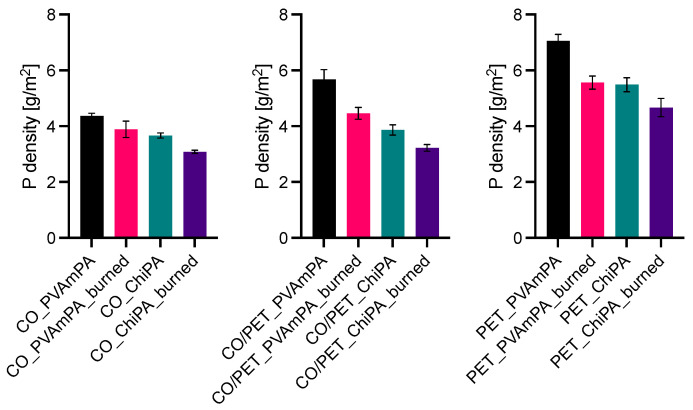
Phosphorus density of coated and burned textiles. Results were obtained using ICP-OES.

**Figure 18:**
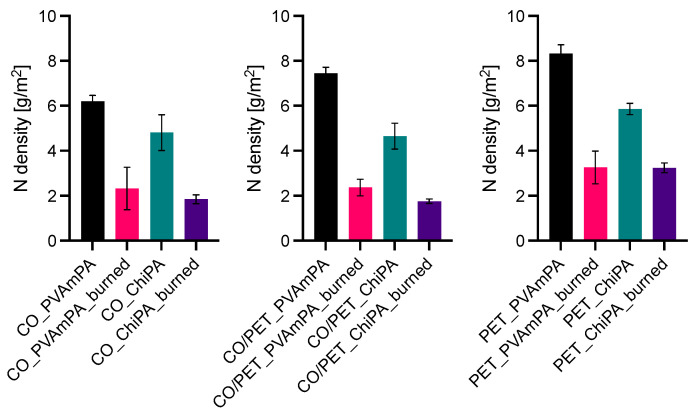
Nitrogen density of coated and burned textiles. Results were obtained using Kjeldahl analysis.

The phosphorus and nitrogen densities of coated and burned textiles are shown in Figure 17 and Figure 18. Phosphorus surface densities were determined by ICP-OES, while nitrogen was quantified via Kjeldahl analysis.

**Figure 19:**
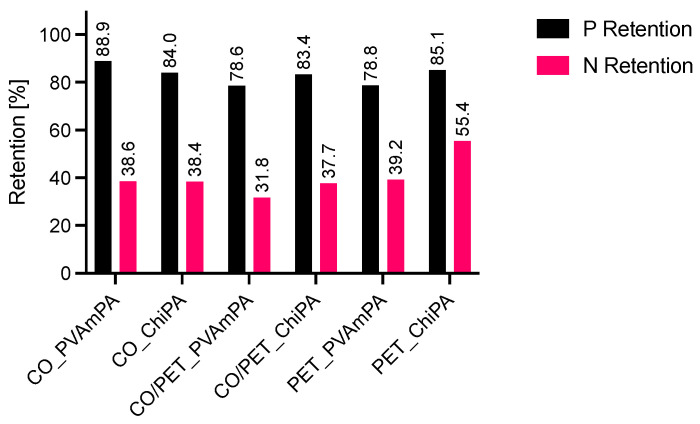
Phosphorus and nitrogen retention of burned textiles.

Across all substrates, PVAmPA-finished fabrics exhibit higher phosphorus surface densities than the corresponding ChiPA-finished samples, both before and after flame exposure. This trend is consistent with the higher phosphorus mass fraction measured for PVAmPA compared with ChiPA and indicates a higher P loading of the applied finish. To quantify the stability of the elemental loadings during flame exposure, retention values were calculated as the ratio of surface density after burning to that before burning (P- and N-retention). For all coated samples, phosphorus retention is below 100%, indicating that a fraction of phosphorus-containing species is released during pyrolysis (Figure 19).

In principle, some phosphorus FRs can contribute in the gas phase via P-containing radicals (e.g., PO, PO_2_, HPO), which slow down combustion by scavenging highly reactive H, O, and OH radicals [59,60]. Py-GC/MS did not yield identifiable P-containing peaks, suggesting that phosphorus-containing species can occur at very low concentrations in complex pyrolysate mixtures and generate weak signals, potentially falling below the detection limit. In the FTIR fingerprint region, phosphate-related P–O vibrations overlap strongly with carbohydrate/ester C–O bands, limiting specificity and preventing the reliable assignment of phosphorus-containing gas-phase products. However, together with the measured P-retention values (<100% after burning), it can be concluded that a portion of phosphorus is released during flame exposure.

Nitrogen surface densities follow the same overall ranking, with higher values for PVAmPA-finished textiles reflecting the higher nitrogen content of the PVAm-based system. After burning, nitrogen loadings decrease, indicating that nitrogen-containing components are at least partially released as volatile species.

**Figure 20:**
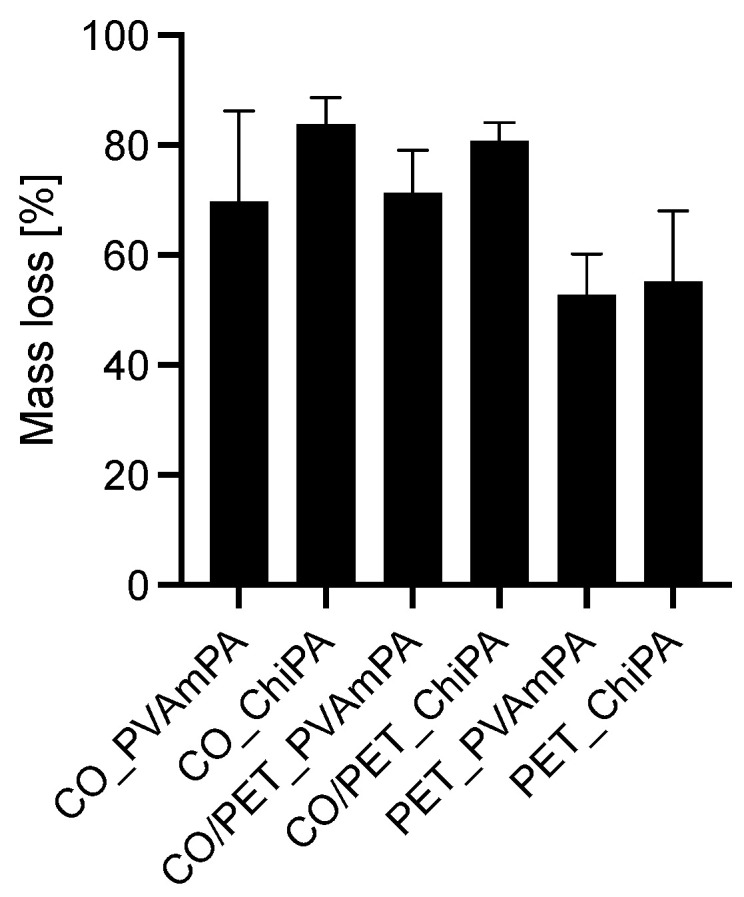
Mass loss of finished textiles.

Figure 20 compares the mass loss after flame exposure for the finished fabrics and therefore provides a simple, integral measure of how much material is consumed during the flame exposure of the circular samples.

Overall, the CO and CO/PET-based fabrics exhibit higher mass losses than the PET fabrics. This means that particularly for finished PET, material consumption is decreased during flame exposure. The mass loss data should be interpreted referring to the substrate-dependent coating thickness and the phosphorus and nitrogen content of the coating. PET forms a substantially thicker surface layer with a higher P/N content, which may simultaneously increase the amount of finish available for volatilization during flame exposure and enhance barrier effects that limit substrate consumption. In contrast, the thinner coatings on CO and CO/PET likely penetrate into the porous fiber, reducing the surface layer mass while increasing interfacial contact that promotes phosphate-driven dehydration and residue formation. Differences between PVAmPA and ChiPA are comparatively small but suggest a tendency toward higher mass loss for ChiPA, which aligns with its generally weaker condensed-phase residue promotion compared with PVAmPA.

## 4. Conclusions

This study demonstrates that a single-step, binder-assisted coating route can transfer phytic acid-derived phosphorus–nitrogen chemistry onto different textile substrates (CO, PET, CO/PET) and achieve measurable flame-retardant effects while also revealing substrate- and formulation-dependent limitations.

Across all three fabrics, both phytate systems achieved effective surface flame suppression in the DIN EN ISO 15025 surface ignition test. In the more application-oriented DIN 75200 burning rate test, PVAmPA provided the most robust overall performance, reducing the burning rate on the coated side from 152 to 0 mm/min for CO and from 171 to 0 mm/min CO/PET. For PET, no hole formation or dripping was observed for either finish under the applied flame exposure conditions.

Performance differences between the two finishes are consistent with their elemental loading and nitrogen availability per unit mass. PVAmPA exhibits higher P/N contents on the fabrics, which correlates with its stronger and more consistent flame-retardant properties. Performance and durability were also influenced by substrate-dependent coating distribution.

Thermal analysis supported a condensed-phase contribution for both finishes. On all substrates, PVAmPA and ChiPA shifted the temperature of 5% mass loss (T_Δ5%_) to lower temperatures and increased the residue at 700 °C relative to untreated fabrics. On CO, the residue increased from 4.5% to 18.2% for PVAmPA and to 15.2% for ChiPA. In MCC analyses, PVAmPA caused the strongest reduction in HRC, particularly for CO/PET, where HRC decreased from 250.9 to 167.8 J/(g·K). ChiPA showed a more mixed behavior (e.g., strong HRC reduction on PET but less favorable THR changes). Gas-phase analyses further indicated dehydration-related volatile release for both finishes and additional NH_3_ evolution for PVAmPA-treated samples.

Wash durability remains the central practical bottleneck between the two systems. Gravimetric wash-off showed systematically higher losses for ChiPA-coated fabrics, while elemental analysis indicated that the retention of P/N-containing species depends strongly on both the substrate and FR system. This suggests that add-on alone is not a reliable descriptor of laundering stability for these binder-assisted coatings and that improved fixation through the optimization of the binder and FR compatibility or stronger anchoring strategies will be required to achieve durability under more demanding laundering conditions.

Overall, the results support the feasibility of a scalable, single-step phytate finish that can deliver meaningful flame-retardant performance across CO, PET, and CO/PET. Future work should prioritize binder optimization to increase FR efficiency while reducing the combustible polymer fraction. In particular, systematic variation in binder chemistry, the FR-to-binder ratio, and curing conditions should be used to improve the fixation and laundering stability of the flame-retardant species on CO, PET, and CO/PET. Since the present results indicate substrate-dependent differences in coating distribution and retention, these effects should be investigated in more detail. In addition, phosphorus-specific analytical methods are needed to better distinguish condensed-phase and gas-phase contributions and to support the development of more durable, application-relevant formulations.

## Figures and Tables

**Table 1:** Formulations for the flame-retardant coating of textiles.

Flame Retardant	m (Lefasol 214/2) g	m (Water) g	m (FR) g	m (BYK 349) g
PVAmPA	66	0	18	0.33
ChiPA	66	6	18	0.33

**Table 2:** Phosphorus and nitrogen mass fraction and molar P/N ratio of PVAmPA and ChiPA.

Flame Retardant	*w*(P) ^1^ %	*w*(N) ^2^ %	P/N Molar Ratio mol/mol
PVAmPA	15.5	8.2	1.0:1.2
ChiPA	9.9	4.2	1.0:0.9

^1^ Measured quantitatively by ICP-OES. ^2^ Measured quantitatively by Kjeldahl nitrogen determination.

**Table 3:** The thermogravimetric analysis data of the flame retardants and their educts.

Sample	Res_700°C_ %	T_Δ5%_ °C	T_max_ (Res) °C (%)
Phytic Acid	38.5	146	335 (76.8) 559 (54.9)
Lupamin 9095	60.7	261	230 (97.6) 349 (87.2) 432 (73.6) 530 (64.9)
Chitosan	34.9	213	226 (80.7)
PVAmPA	43.8	254	269 (93.3) 313 (84.5) 419 (67.7) 479 (56.1) 502 (52.1)
ChiPA	52.6	213	240 (86.7) 301 (70.1) 289 (72.6)

**Table 4:** Thermogravimetric analysis data of coated and uncoated textiles.

Sample	Res_700°C_ %	T_Δ5%_ °C	T_max_ (Res) °C (%)
CO	4.5	330	391 (41.9)
CO_PVAmPA	18.2	320	280 (97.8) 356 (72.3) 418 (31.0)
CO_ChiPA	15.2	291	241 (97.7) 351 (64.7) 427 (26.3)
CO/PET	11.3	341	386 (71.9) 455 (31.7)
CO/PET_PVAmPA	20.4	321	316 (95.8) 353 (83.2) 419 (52.4)
CO/PET_ChiPA	14.6	289	245 (98.0) 347 (79.9) 437 (43.8)
PET	16.0	421	456 (54.8)
PET_PVAmPA	21.5	350	316 (96.8) 415 (63.8) 443 (38.4)
PET_ChiPA	17.3	280	237 (97.7) 437 (55.8) 450 (49.7)

**Table 5:** The microscale combustion calorimetry data of the coated and uncoated textiles.

Sample	HRC J/(gK)	THR kJ/g	T_pkHRR_ °C
CO	208.4 ± 13.1	10.4 ± 0.3	377 ± 3
CO_PVAmPA	172.8 ± 6.5	10.5 ± 0.2	340 ± 2 400 ± 6
CO_ChiPA	224.5 ± 5.1	10.9 ± 0.4	338 ± 2 412 ± 0
CO/PET	250.9 ± 1.5	14.6 ± 0.3	357 ± 4 430 ± 2
CO/PET_PVAmPA	167.8 ± 3.9	12.8 ± 0.2	271 ± 1 352 ± 12
CO/PET_ChiPA	217.7 ± 9.2	14.0 ± 0.7	314 ± 13 415 ± 4
PET	412.7 ± 9.0	14.8 ± 0.2	449 ± 1
PET_PVAmPA	250.5 ± 6.1	14.6 ± 0.6	413 ± 0
PET_ChiPA	221.7 ± 14.0	16.2 ± 0	426 ± 2

**Table 6:** Flame test results according to DIN 75200 for coated and uncoated textiles. Results were compared to maximum allowed burning rate of 102 mm/min as per FMVSS 302.

Sample	Side Facing Flame	PVAmPA	ChiPA	Untreated
CO	Coated Uncoated	0 35	31 55	152
CO/PET	Coated Uncoated	0 66	0 89	171
PET	Coated Uncoated	0 0	0 128	0

## Data Availability

The original contributions presented in this study are included in the article. Further inquiries can be directed to the corresponding authors.

## Supplementary Materials

The following supporting information can be downloaded at https://www.mdpi.com/article/10.3390/polym18070819/s1, Figure S1: Baseline-corrected and normalized FTIR spectra of TGA peak maxima, measured under nitrogen at 20 K/min; Table S1: Comparison of Py-GC/MS results for CO-based textiles with and without flame-retardant treatments; Table S2: Comparison of Py-GC/MS results for PET-based textiles with and without flame-retardant treatments; Table S3: Comparison of Py-GC/MS results for CO/PET-based textiles with and without flame-retardant treatments; Figure S2: Comparison of Py-GC/MS results for all textiles; Figure S3: Total ion chromatograms (TICs) from Py-GC/MS analyses of CO/PET-based textiles with and without flame-retardant treatment; Figure S4: Total ion chromatograms (TICs) from Py-GC/MS analyses of PET-based textiles with and without flame-retardant treatment.

## Appendix A

**Table A1:** Complementary microscale combustion calorimetry data.

Sample	HRC J/(gK)	pk1HRR W/g	pk1HR kJ/g	T_pk1HRR_ °C	pk2HRR W/g	pk2HR kJ/g	T_pk2HRR_ °C
CO	208.4 ± 13.1	208.4 ± 13.1	10.4 ± 0.3	377 ± 3			
CO_PVAmPA	172.8 ± 6.5	102.4 ± 0.6	6.4 ± 0.9	340 ± 2	70.4 ± 7.1	4.2 ± 1.1	400 ± 6
CO_ChiPA	224.5 ± 5.1	123.1 ± 4.3	6.1 ± 0.5	338 ± 2	101.4 ± 0.8	4.8 ± 0.1	412 ± 0
CO/PET	250.9 ± 1.5	94.6 ± 1.1	5.6 ± 0.5	357 ± 4	156.3 ± 2.6	9.0 ± 0.2	430 ± 2
CO/PET_PVAmPA	167.8 ± 3.9	10.4 ± 4.1	0.5 ± 0.2	271 ± 1	29.9 ± 0.8	3.1 ± 0.5	352 ± 12
CO/PET_ChiPA	217.7 ± 9.2	36.3 ± 3.2	2.4 ± 0.1	314 ± 13	181.4 ± 6.0	11.6 ± 0.6	415 ± 4
PET	412.7 ± 9.0	412.7 ± 9.0	14.8 ± 0.2	449 ± 1			
PET_PVAmPA	250.5 ± 6.1	250.5 ± 6.1	14.6 ± 0.6	413 ± 0			
PET_ChiPA	221.7 ± 14.0	221.7 ± 14.0	16.2 ± 0	426 ± 2			
