# Supplementary material for: Single-Step Phytate Flame-Retardant Coatings for Cotton, Polyester and Cotton/Polyester Blends
Source: Polymers (Basel). 2026 Mar 27;18(7):819. doi: 10.3390/polym18070819 (PMC13074638; doi:10.3390/polym18070819)
Supplement: Supplementary file 1 [file polymers-18-00819-s001.zip › polymers-4171072-supplementary.pdf]

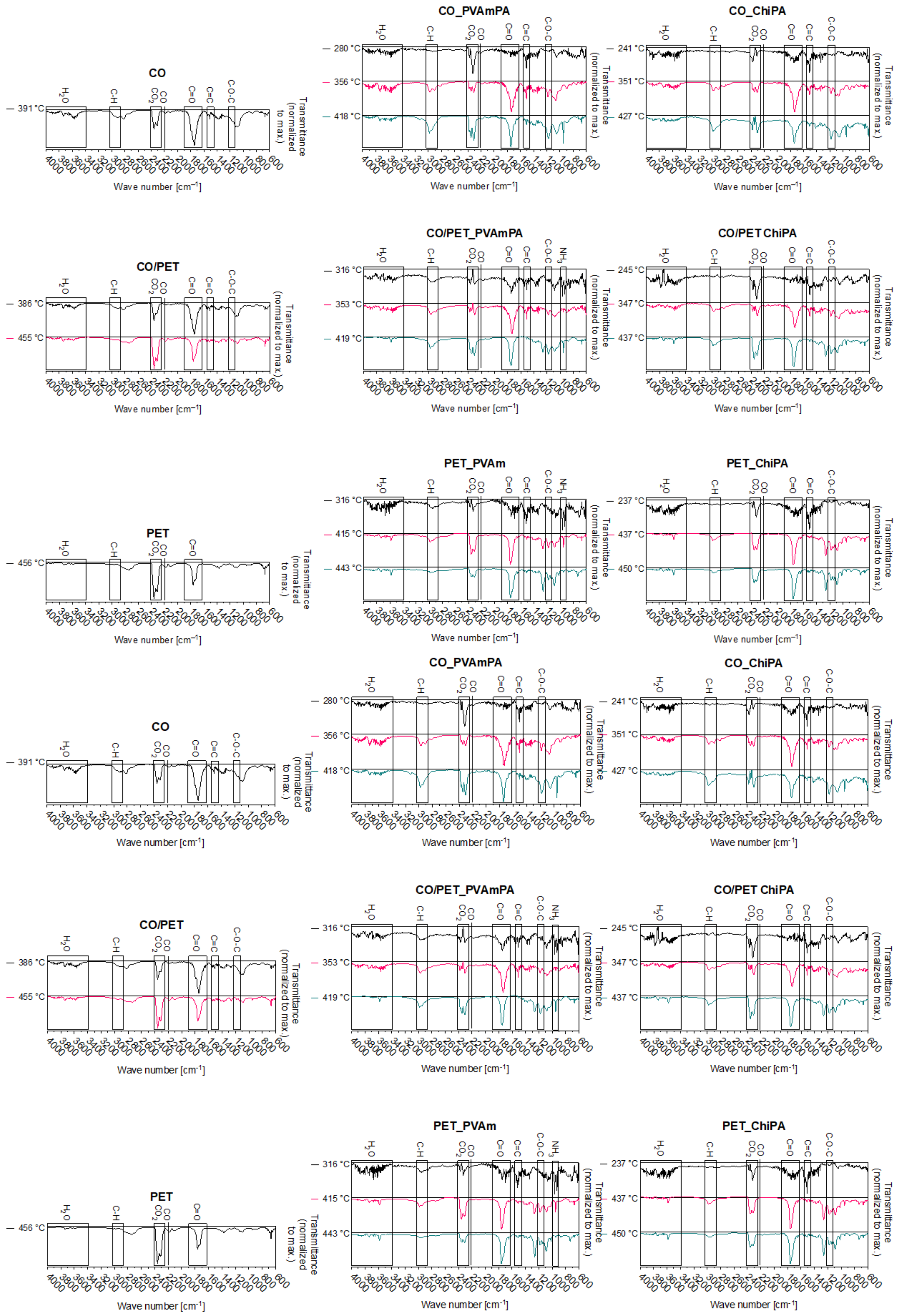

Figure S1. Baseline-corrected and normalized FTIR spectra of TGA peak maxima, measured under nitrogen at 20 K/min.

Table S1. Comparison of Py-GC/MS results for CO-based textiles with and without flame-retardant treatments.

Substance Class	*Relevant Compounds*	*Uncoated CO*	*CO_Binder*	*CO_PVAmPA*	*CO_ChiPA*
Oxygen-Containing Compounds	CO_2	+	+	+	+
	Ethyl acrylate	-	+	+	+
	Hydroxyacetone	+	+	-	-
	2-Furfural	+	+	+	+
	2-Furancarboxaldehyde, 5-methyl-	-	-	+	+
	2-Ethyl-1-butanol	+	-	-	-
	Levoglucosenone	-	-	+	+
	1,4:3,6-Dianhydro-α-d-glucopyranose	+	-	+	+
	5-Hydroxymethylfurfural	+	+	+	+
	Ethyl Hydrogen Glutarate	-	+	+	+
	Pentanedioic acid, 2-methylene-, diethyl ester	-	+	+	+
	Levoglucosan	+	+	+	+
	1,6-Anhydro-β-D-glucofuranose	-	-	-	+
	Triethyl hex-5-ene-1,3,5-tricarboxylate (trimer)	-	+	+	+
Nitrogen-Containing Compounds	Carbonocyanidic acid, ethyl ester	-	+	+	+
	Pyridine, 2-methyl-	-	-	+	-
	2,6-Lutidine	-	-	+	-
	4(1H)-Pyrimidinone	-	+	+	+
	Glutarimide	-	-	-	+

- Not detectable/ extremely weak

+ Detected

Table S2. Comparison of Py-GC/MS results for PET-based textiles with and without flame-retardant treatments.

Substance Class	*Relevant Compounds*	*Uncoated PET*	*PET_Binder*	*PET_PVAmPA*	*PET_ChiPA*
Oxygen-Containing Compounds	CO_2	+	+	+	+
	Ethyl acrylate	-	+	+	+
	1-Hexanol, 2-ethyl-	-	-	+	+
	Pentanedioic acid, diethyl ester	-	+	+	+
	Pentanedioic acid, 2-methylene-, diethyl ester	-	+	+	+
	Triethyl hex-5-ene-1,3,5-tricarboxylate (trimer)	-	+	+	+
Aromatic Compounds	Benzene	+	-	+	+
	Styrene	-	-	+	+
	Acetophenone	-	-	+	+
	Vinyl Benzoate	+	+	+	+
	Benzoic Acid	+	+	+	+
	Divinyl terephthalate	+	+	+	+
	1,3-Benzenedicarboxylic acid, diethyl ester	-	+	-	-
Nitrogen-Containing Compounds	Pyridine, 2-methyl-	-	-	+	+
	2,6-Lutidine	-	-	+	-
	Benzonitrile	-	-	+	+
	Benzene, 1-isocyano-3-methyl-	-	-	+	-
Condensed Aromates/ Higher Fragments	Biphenyl	+	-	+	+
	(1,1'-Biphenyl)-2,2'-dicarboxaldehyde	+	-	-	-
	Ethylene dibenzoate	+	-	-	-

- Not detectable/ extremely weak
+ Detected

Table S3. Comparison of Py-GC/MS results for CO/PET-based textiles with and without flame-retardant treatments.

Substance Class	*Relevant Compounds*	*Uncoated CO/PET*	*CO/PET_Binder*	*CO/PET_PVAmPA*	*CO/PET_ ChiPA*
Oxygen-Containing Compounds	CO_2	+	+	+	+
	Ethyl acrylate	-	+	+	+
	Hydroxyacetone	+	-	-	-
	Methyl pyruvate	+	-	-	-
	2-Furfural	-	-	+	-
	Levoglucosenone	-	-	+	+
	Pentanedioic acid, diethyl ester	-	+	+	+
	Pentanedioic acid, 2-methylene-, diethyl ester	-	+	+	+
	Levoglucosan	+	+	+	+
	Triethyl hex-5-ene-1,3,5-tricarboxylate (trimer)	-	+	+	+
Aromatic Compounds	Vinyl Benzoate	+	-	-	-
	Benzoic Acid	+	+	+	+
	1,2-Ethanediol, monobenzoate	-	-	+	-
	Divinyl terephthalate	+	+	+	+
	1,3-Benzenedicarboxylic acid, diethyl ester	-	+	-	-
Nitrogen-Containing Compounds	Carbonocyanidic acid, ethyl ester	-	-	+	-
	Pyridine, 2-methyl-	-	-	+	-
	2,6-Lutidine	-	-	+	-
	Benzonitrile	-	-	+	-
	Benzene, 1-isocyano-3-methyl-	-	-	+	-
Condensed Aromates/ Higher Fragments	Biphenyl	+	-	+	-
	Ethylene dibenzoate	+	-	-	-

- Not detectable/ extremely weak

\+ Detected

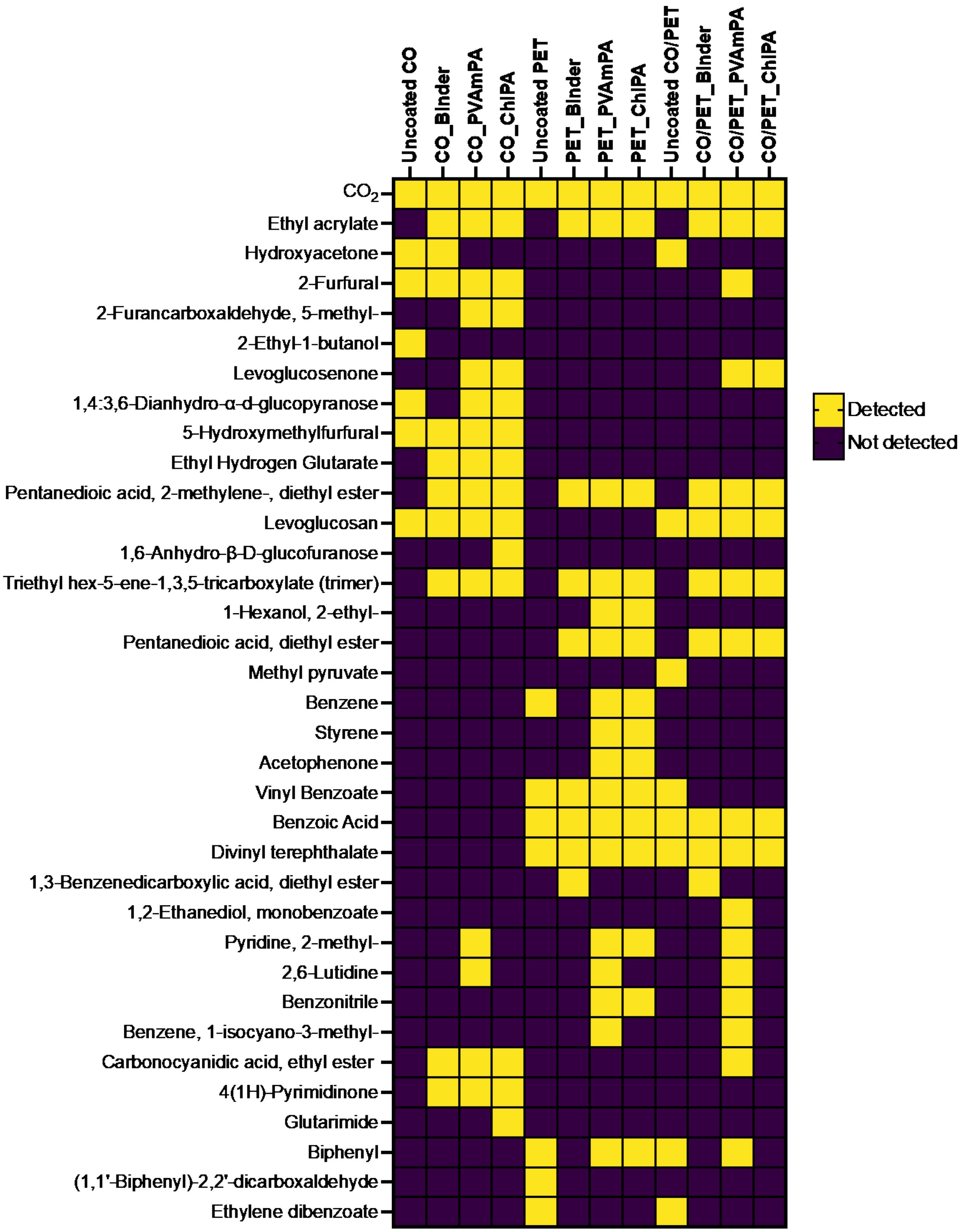

Figure S2. Comparison of Py-GC/MS results for all textiles.

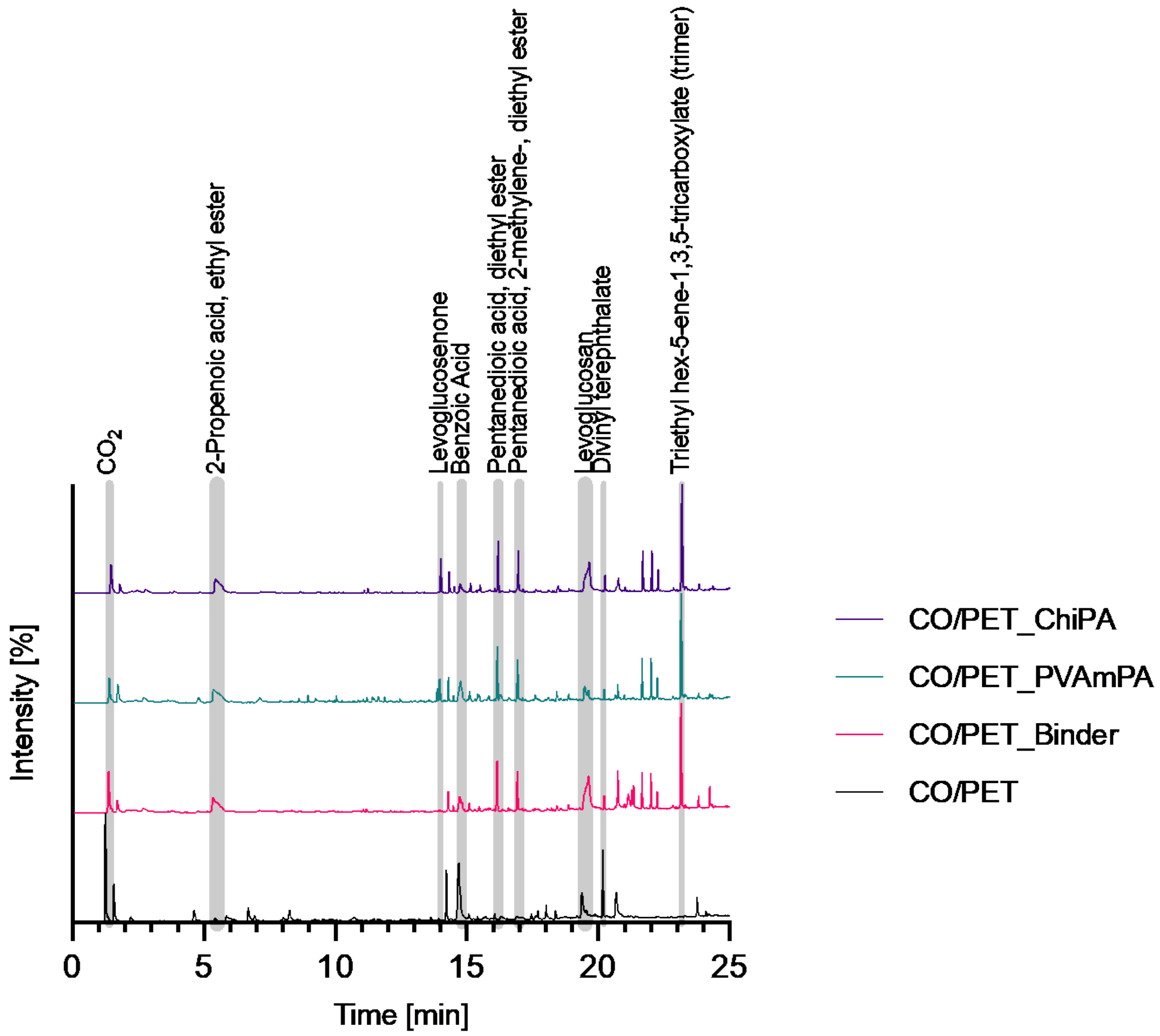

Figure S3. Total ion chromatograms (TICs) from Py-GC/MS analyses of CO/PET-based textiles with and without flame-retardant treatment.

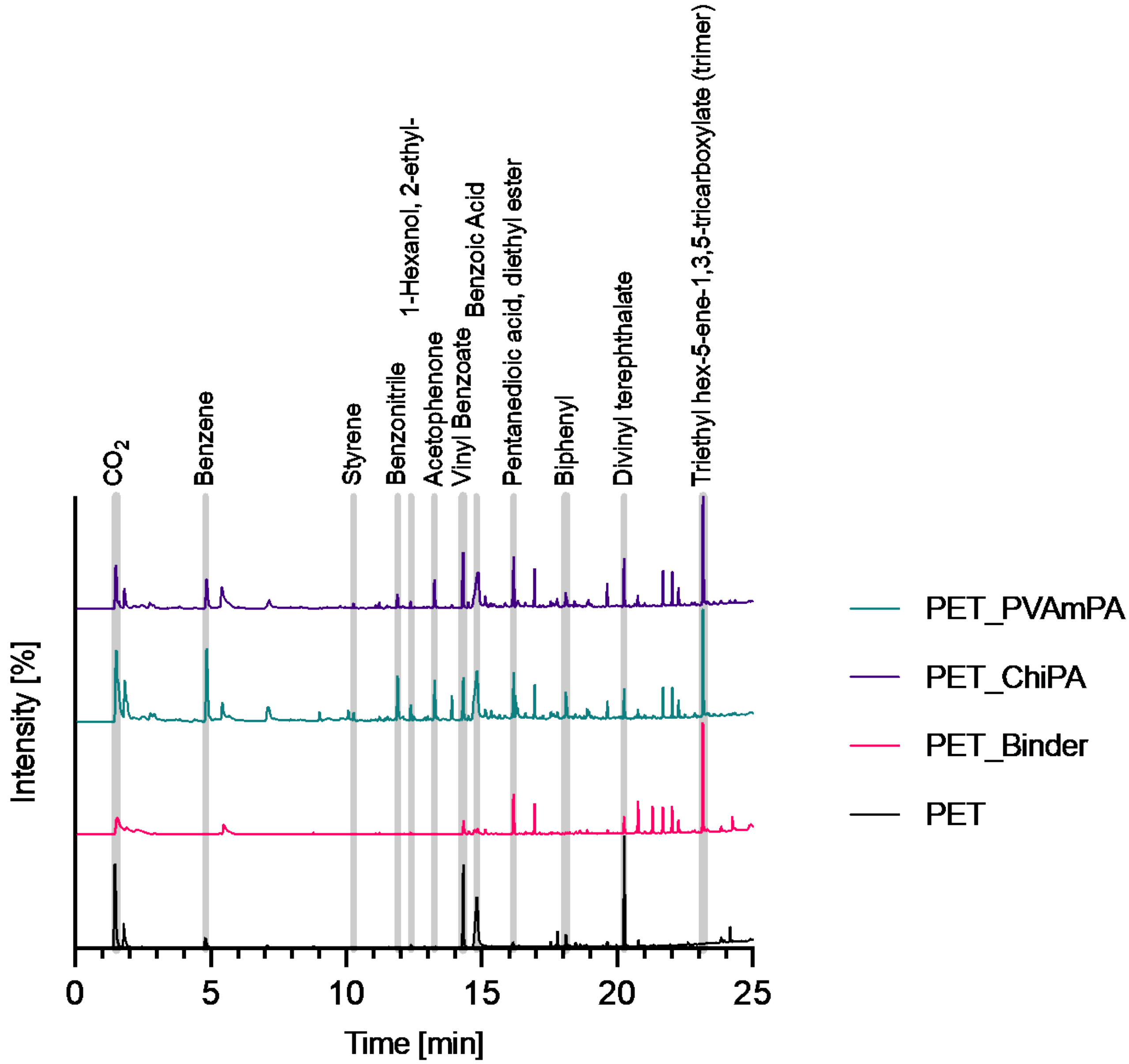

Figure S4. Total ion chromatograms (TICs) from Py-GC/MS analyses of PET-based textiles with and without flame-retardant treatment.
